# *“*Saying goodbye all alone with no close support was difficult”- Dying during the COVID-19 pandemic: an online survey among bereaved relatives about end-of-life care for patients with or without SARS-CoV2 infection

**DOI:** 10.1186/s12913-021-06987-z

**Published:** 2021-09-22

**Authors:** Karlotta Schloesser, Steffen T Simon, Berenike Pauli, Raymond Voltz, Norma Jung, Charlotte Leisse, Agnes van der Heide, Ida J Korfage, Anne Pralong, Claudia Bausewein, Melanie Joshi, Julia Strupp, for PallPan and the CO-LIVE study

**Affiliations:** 1 Department of Palliative Medicine, Faculty of Medicine and University Hospital, University of Cologne, Kerpener Street 62, 50937 Cologne, Germany; 2 Center for Integrated Oncology Aachen Bonn Cologne Dusseldorf (CIO ABCD), Faculty of Medicine and University Hospital, University of Cologne, Cologne, Germany; 3 Clinical Trials Center (ZKS), Faculty of Medicine and University Hospital, University of Cologne, Cologne, Germany; 4 Center for Health Services Research. Faculty of Medicine and University Hospital, University of Cologne, Cologne, Germany; 5 Department I of Internal Medicine, Faculty of Medicine and University Hospital, University of Cologne, Cologne, Germany; 6 Department of Public Health, University Medical Center Rotterdam, Erasmus, MC the Netherlands; 7 Department of Palliative Medicine, LMU University Hospital Munich, Munich, Germany; 8 Comprehensive Cancer Centre Munich (CCCM), Munich, Germany

**Keywords:** Communication, Palliative care, SARS-CoV2, Relatives, Dying, Support, Post-bereavement survey, Visiting restrictions

## Abstract

**Background:**

During the SARS-CoV2 pandemic, protection measures, as well as visiting restrictions, had a severe impact on seriously ill and dying patients and their relatives. The study aims to describe the experiences of bereaved relatives of patients who died during the SARS-CoV2 pandemic, regardless of whether patients were infected with SARS-CoV2 or not. As part of this, experiences related to patients’ end-of-life care, saying goodbye, visiting restrictions and communication with the healthcare team were assessed.

**Methods:**

An open observational post-bereavement online survey with free text options was conducted with 81 bereaved relatives from people who died during the pandemic in Germany, with and without SARS-CoV2 diagnosis.

**Results:**

67/81 of the bereaved relatives were female, with a mean age of 57.2 years. 50/81 decedents were women, with a mean age of﻿ 82.4 years. The main underlying diseases causing death were cardiovascular diseases or cancer. Only 7/81 of the patients were infected with SARS-CoV2. 58/81 of the relatives felt burdened by the visiting restrictions and 60/81 suffered from pandemic-related stress. 10 of the patients died alone due to visiting restrictions. The burden for relatives in the hospital setting was higher compared to relatives of patients who died at home. 45/81 and 44/81 relatives respectively reported that physicians and nurses had time to discuss the patient’s condition. Nevertheless, relatives reported a lack of proactive communication from the healthcare professionals.

**Conclusions:**

Visits of relatives play a major role in the care of the dying and have an im﻿pact on the bereavement of relatives. Visits must be facilitated, allowing physical contact. Additionally, virtual contact with the patients and open, empathetic communication on the part of healthcare professionals is needed.

**Trial registration:**

German Clinical Trials Register (DRKS00023552).

## Background

The SARS-CoV2 (severe acute respiratory syndrome coronavirus type 2) pandemic disrupted usual experiences of dying and bereavement for patients and their relatives. To mitigate the devastating impact of SARS-CoV2, patients were isolated in care facilities and died alone as visit restrictions were placed regardless of a SARS-CoV2 infection. Although there were exceptions to restrictions, e.g. allowing limited visits in the dying phase, they still had effects on the dying patient and their relatives [1].

Funerals and burials were postponed or held remotely, often without the presence of relatives or friends. Wallace et al. [2] found that some grief processes during the SARS-CoV2 pandemic were novel related to physical distancing and isolation alongside experiencing uncertainty and self-blame related to infection.

Selman et al. [3] recommended proactive, sensitive, and regular communication with relatives while providing transparent information to alleviate risks of adverse outcomes (e.g., emotional distress) –on patients and relatives – during the pandemic. They suggested enabling relatives to say goodbye in person where possible while also supporting virtual communication, to provide excellent symptom control alongside emotional and spiritual support. Palliative care providers serve as a resource here based on their expertise in end-of-life care, symptom management, communication, counselling, and including relatives as the unit of care [4]. However, due to restrictions on visits, some health care professionals (i.e. physiotherapists, psychologists, social workers) and volunteers were also not allowed to visit and care for patients. This could lead to a discontinuity in care, a lack of communication, poorer patient care, and complicated grief [5–7]. This underpins that the core values of palliative care were impacted negatively by the SARS-CoV2 related policies and practices. Providing adequate and compassionate care for the dying and their relatives were difficult – although being a fundamental human right [8].

This will likely have affected the experience of the dying and their relatives. Up to date, it is unknown what the intermediate and longer effects of these experiences will be. Recently published findings from the UK [9–11] describe the relatives’ need of visiting the seriously ill and dying patient, despite visiting restrictions. Here, health care professionals are faced with the challenge to recognise the dying phase early enough to allow the relatives to be present before death [10, 11]. In the absence of having the opportunity to visit, the importance of connecting virtually with the patient increases. Health and social care professionals can mitigate the absence of relatives’ visits at the end of life by providing alternative ways of saying goodbye to the patient or enabling virtual connectedness [9].

Given the limited data so far, understanding the impact of the current SARS-CoV2 pandemic is pertinent to provide better support for dying patients and their relatives. Our study aimed to describe the experiences of bereaved relatives of patients who died during the SARS-CoV2 pandemic in Germany related to patients’ end-of-life care, saying goodbye, visiting restrictions, and communication with the healthcare team. Results will also be used to support the development of a national strategy for the care of severely ill and dying patients and their relatives for better pandemic preparedness and response.

## Methods

### Study design

 The study is part of a German collaborative project entitled “National Strategy for Palliative Care of Severely Ill and Dying People and their Relatives in Pandemics (PallPan) in Germany,“ led by the National Research Network of University Medicine (NUM) on COVID-19. PallPan aims to develop and consent a national strategy for the care of seriously ill and dying patients to ensure future pandemic preparedness. Additionally, this study was conducted jointly with the CO-LIVE-study [12] on end-of-life care practices as provided during the first peak of the pandemic in different European healthcare settings. Results from Germany are presented.

We conducted an open observational post-bereavement survey to assess relatives´ experiences with end-of-life care during the SARS-CoV2 pandemic. An online survey using LimeSurvey was developed that enabled responses via computer or mobile devices. The study was conducted following the Checklist for Reporting Results of Internet E-Surveys (CHERRIES) guidelines [13] and approved by the local Research Ethics Committee of the University of Cologne (No. 19-1456-3; 27.04.2020). The research was conducted according to the Declaration of Helsinki.

### Survey Development and Pre-Test

The questionnaire included an abbreviated version of the international Care Of the Dying Evaluation (iCODE) questionnaire that focuses on the last two days of life and the bereavement period, and asks about the characteristics of patient care and family support[14]. It included also the Positive and Negative Affect Schedule (PANAS) with two subscales, each consisting of 10 items: positive affect and negative affect [15, 16]. We added self-developed questions about the impact of SARS-CoV2 associated measures: symptom control of pain and other symptoms, communication with the healthcare team, emotional and spiritual support, and circumstances surrounding death, support from others, education in hygiene, and the provision of protective equipment. For suggestions and experiences, relatives were invited to enter free text comments. A final question asked for further comments on supporting bereaved people during the pandemic.

There were no incentives for participation, and respondents could discontinue the survey at any time. The survey was piloted with 20 adults from July 15th to July 21st 2020 and modified accordingly.

### Data collection

The online survey was open from July 21st to November 15th 2020.

The questionnaire could be completed by all bereaved people in Germany whose relative had died between March 1st and November 15th 2020 at home, in a hospital, a nursing home, hospice or elsewhere, with or without infection with SARS-CoV2. Visiting restrictions in care facilities[17, 18] have been placed between March 13th and May 10th 2020. The survey was advocated through relevant networks, e.g. PallPan, using the snowball technique, and an article in a regional newspaper[19]. Participants were fully informed about the study and provided electronic written informed consent before starting the online survey. All responses were anonymous and allocated a response number for analysis.

### Data analysis

Descriptive data analysis was performed by a group of researchers and clinicians. IBM SPSS 27 was used and results are presented as mean ± SD and count (percentage), respectively. The data is presented under the topics that also guided the different questions within the survey. Subgroup analyses based on SARS-CoV2 infection, place of death and visiting ban were conducted. Free text comments were analysed using a content analysis approach.

## Results

### Survey sample

**Fig. 1:**
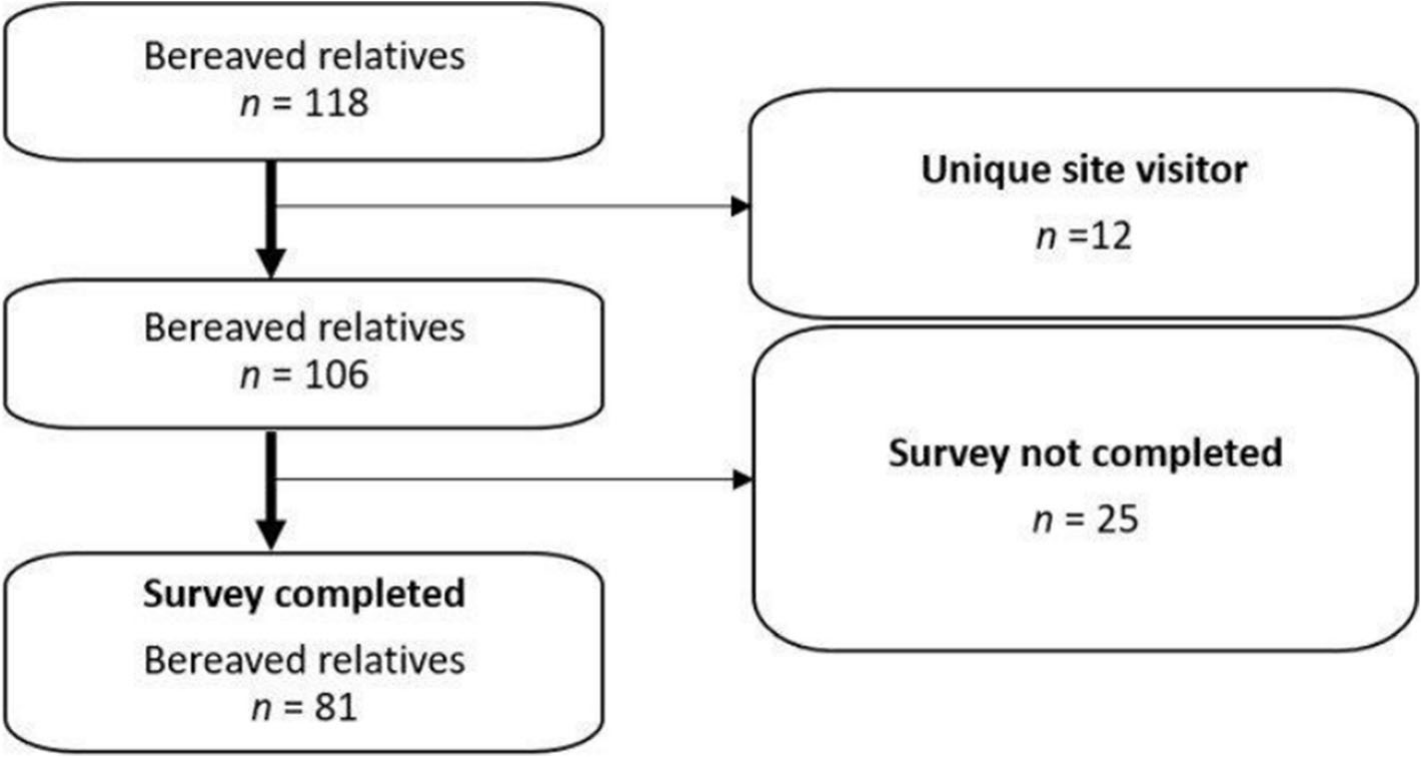
Flow chart depicting the survey participants

118 bereaved relatives visited the survey site. 12 were identified as unique site visitors an﻿d further 25 did not complete the survey (participation rate: 89 %). The analysis is based on the 81 completers (completion rate: 76 %, Fig. 1). Bereaved relatives had the opportunity to provide free-text comments (the number in parentheses indicates the number of comments) on the topics of *pandemic-related limitations in end-of-life care* (*n* = 67), *greatest challenges in end-of-life care due to the pandemic* (*n* = 74), *pandemic-related stress* (*n* = 54), *wishes for end-of-life care in pandemic times* (*n* = 63), *ideas and suggestions on how to better care for patients in the last phase of life during a pandemic* (*n* = 68) and *other comments* (*n* = 56). Since relatives repeated the same themes consistently regardless of the question, the free text fields would be evaluated across questions and considered based on the predominant themes (e.g. visiting restrictions).

Characteristics of the decedents and the bereaved relatives are presented in Table 1.

**Table 1:** Demographics and characteristics of bereaved relatives and decedents. (*n* = 81)

*Decedents*	*n (%) or* mean *(SD)*		*Bereaved relatives*	*n* (%) or mean (SD)	
**Age** (in years)	82.4	(12)	**Age** (in years)	57.2	(12)
**Gender**			**Gender**		
female	50	(62)	female	67	(83)
male	31	(38)	Male	13	(16)
divers	0		Divers	1	(1)
**SARS-COV2 infection**^**a**^			**SARS-CoV2 infection**^**a**^		
yes	7	(9)	yes	1	(1)
no	73	(90)	no	75	(93)
unknown	1	(1)	unknown	5	(6)
**Reason for death**			**Relationship to the deceased person**		
cancer	20	(25)	child	52	(64)
cardiovascular disease	18	(22)	partner	14	(17)
diabetes mellitus	1	(1)	sister	3	(4)
dementia	2	(3)	aunt	2	(3)
COVID-19	4	(5)	grandfather	2	(3)
another respiratory disease	1	(1)	other	8	(10)
other	35	(43)			

^a^ Bereaved relatives asked about the decedents SARS-COV2 infection had the option to chose *yes/no probably infected*; *yes/no infected (tested); unknown*. *yes/no* *probably/tested infected* is presented summarized as *yes/no*.

### Visiting restrictions and end-of-life companionship during the SARS-CoV2 pandemic

#### Burdens by visiting restrictions

58/81 (72 %) of the bereaved relatives felt burdened by the visit restrictions of the dying patient and 60/81 (74 %) relatives experienced pandemic-related stress. Patients died between March and October and a third died in general hospitals (30/81; 37 %) or nursing homes (27/81; 33 %). Almost every tenth patient died at home (9/81; 11 %) or in a hospice (8/81; 10 %). 72/81 (89 %) died in care facilities, 6 relatives provided contradictory answers regarding visiting restrictions (no visits and no visit restrictions ticked) and were excluded. There were 23/66 (35 %) unrestricted visiting opportunities, compared to 17/66 (26 %) cases where no visits were allowed. In 58/66 (88 %) cases visits were possible but restricted. If there were visiting restrictions, most of these relatives were allowed to stay for one hour or/and to visit alone. 34/81 (42 %) patients died alone, 10/34 (29 %) died alone due to visiting restrictions. 53/81 (65 %) of the bereaved relatives judged that the decedent died in the right place.

In the free-text comments, the relatives reported: visiting restrictions led to a lack of physical closeness, having to visit alone (no emotional support from others), the missing possibility of end-of-life care provided by relatives, no possibility of saying goodbye, and more organizational effort concerning visits. The visiting restrictions were often only reduced in the dying phase, which was felt to be too late since many patients were no longer responsive at that time to say goodbye.

*It was of course difficult, due to the pandemic, not to be allowed to visit my father in the hospital at first and then only to go to him alone. Saying goodbye to him all alone, without close support was difficult.*

(Bereaved daughter, 53 years, father died in the hospital, he was SARS-CoV2 positive)

*That practically no end-of-life care was possible. Except for the last hours, in which he was only asleep, we were denied access to the nursing facility, which in my opinion was an unbearable situation for both sides.*

(Bereaved daughter, 67 years, father died in the nursing home, he was SARS-CoV2 negative)

*That I was not allowed to accompany my mother [at the end of her life], to know that she must die alone and lonely.*

(Bereaved daughter, mother died in the hospital, she was SARS-CoV2 positive)

#### Impact of the visiting ban on visiting opportunities

**Fig. 2:**
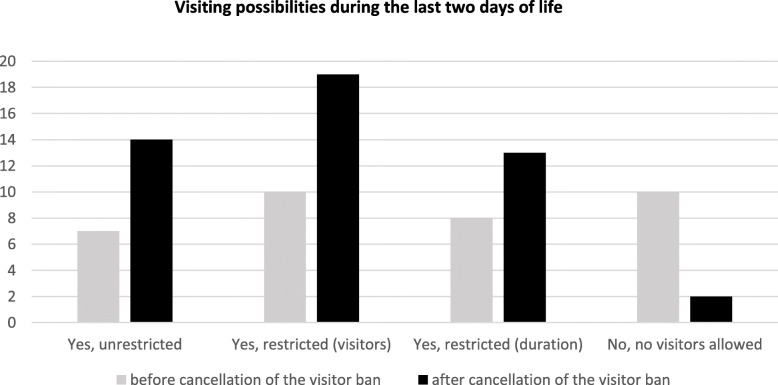
Visiting possibilities (unrestricted visits, visits restricted regarding the number of visitors or the duration; no visitors allowed) during the last two days of life subdivided according to the visit ban at the time of the patients’ death. Frequencies are given; multiple answers possible

Of the 72 patients who died in care facilities, 30 patients died during the official visiting ban and 37 patients died when it was ended. 5 relatives did not know the date of death and 6 relatives provided contradictory answers (no visits and no visit restrictions ticked). Answers from 61 relatives were included in the analysis: 26 relatives who lost the patient during and 35 relatives who lost the patient after the official visiting ban provided information. After ending the official visiting ban in care facilities, two facilities still did not allow visitors (2/35; 6 %; see Fig. 2). Asked for the burden caused by the pandemic situation (additionally to the fact of providing end-of-of-life-care), the visiting ban had a severe impact: Almost all (26/30; 87 %) relatives felt burdened during and slightly more than two thirds (27/37; 73 %) after lifting of the visitor ban.

#### Place of death

**Fig. 3:**
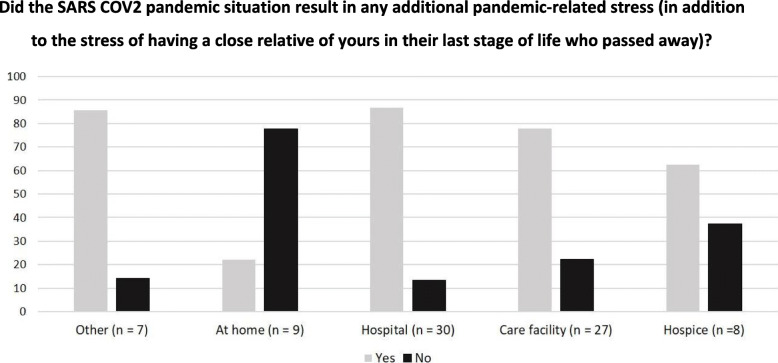
Subgroup analysis: pandemic-related stress relatives suffered in relation to the patient’s place of death. Frequencies are given as percentages, *n* = 81

30/81 (37 %) patients died in a hospital, 27/81 (33 %) died in a care facility, 9/81 (11 %) died at home, 8/81 (10 %) died in a hospice and 7/81 (9 %) patients died elsewhere. All bereaved relatives of persons who died at home indicated that the decedent passed away at the right place (9/9; 100 %). Relatives of patients cared for at home reported feeling less burdened by the pandemic situation (experienced burdened when the patient died at home: *n* = 2/9; 22 % vs. when the patient died in the hospital: *n* = 26/30; 87 %, Fig. 3). Some relatives reported that consequences of the pandemic like short time or no work allowed spending more time with the dying:

*Through Covid-19, I was not able to be employed. This allowed me to fully care for and provide for my loved one. I am grateful for the time we had together to say goodbye. This allowed me to fully care for my loved one. I am grateful for the time we had together to say goodbye. …*.

(Bereaved daughter, 66, father died at home, he was SARS-CoV2 negative)

#### SARS-CoV2 infection

To compare visiting restrictions among patients with/without SARS-CoV2 infection only answers concerning patients who died in care facilities (72/81; 89 %) were included and 6 answers with contradictory information (no visits and no visit restrictions ticked) were excluded. From the resulting 66 cases were 59/66 (89 %) SARS-CoV2 negative and 6/66 (9 %) SARS-CoV2 positive (one relative was not sure about the decedent’s infection status and this data was not included in the analysis). Comparing the visit restrictions among those patients with and without SARS-CoV2 infection, it became apparent, that visit restrictions for SARS-CoV2 positive patients were slightly stricter than for SARS-CoV2 negative patients: in 2/6 (33 %) of the cases were no visits allowed for SARS-CoV2 positive patients and in 12/59 (20 %) of the cases were no visits allowed for SARS-CoV2 negative patients.

Relatives of patients with SARS-CoV2 infection felt more burdened by the pandemic situation compared to those from SARS-CoV2 negative patients: 7/7 (100 %) of the relatives of SARS-CoV2 positive patients answered “yes” to the question about the experience of a pandemic-related burden compared to 52/73 (71 %)of the relatives of SARS-CoV2 negative patients.

#### Online communication with the dying patient

**Fig. 4:**
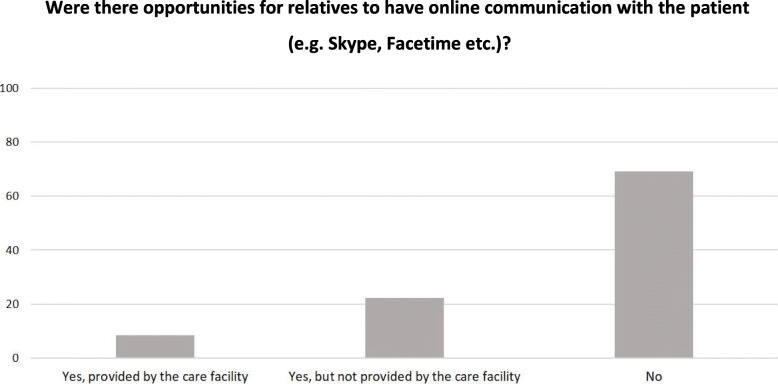
Online communication opportunities for relatives. Frequencies are given as percentages, *n* = 81.

In case of visiting restrictions, opportunities for digital communication (e.g. via Skype) with the patient were limited, irrespectively of the place of death: in 56/81 (69 %) cases no online communication opportunities were given, in 18/81 (22 %) cases the online communication opportunity was given but not provided by the care facility and in 7/81 (9 %) of the cases the online communication was provided by the care facility (Fig. 4).

### Bereaved relatives‘ perspective on communication with the healthcare team during the SARS-CoV2 pandemic

During the last phase of life of the patients, 45/81 (56 %) respectively 44/81 (54 %) relatives reported that physicians resp. nurses had time to listen and discuss the patient’s condition, provided enough information about the patient’s condition (48/81; 59 %) and explanations were easy to understand (61/81; 75 %). 60/81 (74 %) relatives were told that the patient was likely to die soon, but 45/81 (56 %) were not told about what to expect in the dying phase (e.g. symptoms). 56/81 (69 %) relatives assessed the emotional support from the healthcare team as good/excellent, and 61/81 (75 %) felt treated empathically after the death, but 11/81 (14 %) did not have any contact with the healthcare team after the death of the patient.

In the free-text comments concerning the communication with the healthcare team, relatives reported a lack of open, proactive communication (accompanied by a lack of transparency regarding decisions concerning the patient and the treatment, reliable, accessible contact persons and a continuous flow of information) and a deficit of empathy of carers towards the relatives.

*The nursing home had stopped all communication. No consideration was given to contact needs. The nursing staff was completely overworked. The home’s management was disastrous. We were not informed in time about the current situation, we were not involved and we were not consulted about the treatment*.

(Bereaved son, 68 years; father died in a nursing home, he was SARS-CoV2 negative)

## Discussion

### Visiting restrictions

Our study described bereaved relatives’ experiences with end-of-life support of patients who died with or without SARS-CoV2 infection in Germany. Findings revealed that relatives not only had to cope with the stress caused by losing a relative but that they additionally experienced stress directly related to the pandemic, mainly due to the burden caused by visiting restrictions.

Compared to UK data, which report that half of the bereaved relatives (56 %; 10) were not allowed to visit during the last days of the patient’s life, the situation in Germany was more diverse: while one-quarter of the relatives were not allowed to visit *at all*, more than 88 % of the relatives were allowed to visit, but restrictions concerning duration or number of visitors were imposed on them. Common to both studies is the great distress that bereaved relatives experienced due to the visiting restrictions [10]. Those relatives who were able to visit more likely reported a feeling of being adequately supported during the last days of life [10], this – once again- underpins the importance of visits.

Visiting restrictions are at odds with the need of being present when a relative is dying[20, 4, 21, 9] and studies have shown that relatives cope and adjust better in bereavement when they are involved in end-of-life care [22]. Most relatives who were not or only with restrictions allowed to visit their loved ones felt burdened by the situation and described suffering from the lack of physical closeness, saying goodbye and no appropriate end-of-life care, and not knowing the condition of the patient, this is also reported in recent studies [10, 9]. Face-to-face visits are crucial for dying patients[23–25] and accompanying relatives [26–29, 3]. The possibility of face-to-face visits also impacts the processing of the death. Bereaved families in the present study regretted that visiting restrictions were often lifted too late, e.g., when the patient was no longer responsive to say goodbye, this underpins findings from the UK [10]. Identifying the appropriate moment as the end-of-life and thus facilitating visits was reported as a challenge by health care professionals: estimating the remaining time wrong, relatives did not get time to say goodbye to the patient. Healthcare professionals had the impression that relatives would benefit from having the opportunity to visit when the dying patient is conscious and responsive and not just the hours before death [11]. The healthcare professionals’ description enhances the findings of the present study: it is essentially giving relatives and patients the chance to say goodbye early enough to avoid “missing the moment before” death and to say goodbye when the patient is responsive. This is in line with best clinical practice, suggesting that relatives should not only be allowed to visit the dying but also severely ill and deteriorating patients [3, 30, 31]. Also during pandemic situations, relatives should be allowed to visit the deteriorating patients early for saying goodbye and appropriate end-of-life care for both sides. It should be enabled under consideration of necessary hygiene and safety measures. Visiting restrictions caused additional organizational challenges to the accompanying relatives and emotional support by other visiting relatives was missing.

Even after the official visiting ban has been lifted, some facilities still did not allow any visitors, causing an additional burden for relatives. To our knowledge, there are no analyses of what organizational consequences care facilities drew from lifting the visiting ban. In addition, there should be uniform regulations on how visits, apart from a general ban on visits, should be handled.

Relatives of patients who died at home felt less burdened by the pandemic situation than relatives of patients who died in care facilities. They mentioned that the pandemic situation (e.g. flexibility due to home office) was rather beneficial, allowing to spend more time with the patient. Vice versa, Mayland et al. [10] describe that especially relatives who experienced the death in a nursing home or hospital suffered from distress due to visiting restrictions. Being allowed to accompany a dying family member in the last phase of life may be important for the bereavement process [32, 33].

Of the bereaved relatives in our study, those of SARS-CoV2 positive patients felt more burdened and visiting arrangements were stricter. Often, relatives of SARS-CoV2 positive patients are also tested positive, so that quarantine regulations make visits even more difficult. As this project set the focus on palliative care patients irrespectively of a SARS-CoV2 infection, the majority of the decedents and their relatives were SARS-CoV2 negative. Still, they suffered from hygiene and safety measures, and there are signals that visiting arrangements for SARS-CoV2 positive patients were even stricter. Data concerning divergent experiences between SARS-CoV2 negative/positive patients need to be interpreted with great caution due to the descriptive data reported and group sizes differences.

Given the importance of visits at the end of life, visiting regulations and infection control measures should be reconciled to avoid SARS-CoV2 infections and enable contacts. Therefore evidence-based interventions as tracing and isolating SARS-CoV2-positive cases or rapid SARS-CoV2 testing are useful [34, 35]. Facilities should inform about COVID-19 regulations on their websites and guide relatives through their regulations. This contributes to transparent communication and helps relatives to understand the reasons for the regulations [26, 29]. Individual decisions instead of one-size-fits-all rules could help to do justice to the dying patients and their relatives. In December 2020, an order of one state of Germany (North Rhine-Westphalia) decreed that visits must be allowed from the 6th day of an inpatient stay to prevent too long periods of non-face-to-face contact between relatives [36].

When relatives were not allowed to spend face-to-face time with the patient due to the pandemic situation, video or telephone calls could be a way to achieve connectedness. Hanna et al.[11] describe the importance for family members to stay virtually connected with the dying family member in the last phase of life in the absence of visiting: it allows relatives to see for themselves that the patient is *doing okay* and creates a connection between the patient and the usual family life [11]. The present survey showed that only a few facilities provided online communication; the majority of the bereaved relatives were not given the opportunity. Depending on the patient’s cognitive condition and the level of support provided, online communication can be difficult for some patients. Health and social care professionals reported that especially when a patient was suffering from dementia or another cognitive impairment, video calls were rarely offered because of the concern that it might be distressing for the patient or relative [11]. A similar survey from the UK [10] reports fewer relatives/patients who were unable to communicate virtually than the ones in the current survey (UK: 34 % vs. Germany 69 %) and missing equipment as an obstacle in providing online communication [11]. Even with the option for online communication, it cannot compensate for physical contact.

This corresponds to a recent study’s findings that virtual interactions were rare [9, 11] and shows that this area provides significant opportunities for improvement, and holds the possibility to support highly distressed relatives and dying family members.

### Communication with the healthcare team

Our survey revealed mixed experiences of bereaved relatives regarding communication with the healthcare teams. About half of the relatives indicated that the team had enough time to listen and discuss the patient’s condition and had been provided with enough information. Bereaved relatives’ judgment in the present study did differ between doctors and nurses, but data from the UK shows that relatives had a greater level of confidence in the nursing staff and that they provided care with more respect and dignity than doctors [10]. This might be due to differences in the health care system in the two countries (the UK vs. Germany). Three-quarters felt treated empathically by the healthcare team. However, using the free text comments, relatives expressed regret about a lack of open, proactive communication accompanied by a deficit of reliable contact persons and information. Studies underpinned the importance for relatives, especially when they are not allowed to physically visit the dying patient, to be provided with clear and precise information about his/her condition [9]. This comprises information regarding symptom management [37, 38], health decline [38, 39], and personal aspects of care [37]. In this aspect, the relatives’ needs were not met, not only concerning the amount of information but also the way it was provided. This demonstrates the relevance for healthcare teams to install ways to provide reliable information about the patient and to focus on empathic communication. UK patients’ relatives highly valued compassionate care from the healthcare team, allowing early visits before the death of the patient to say goodbye [10].

As the survey relates to the beginning pandemic situation in Germany, healthcare professionals were overwhelmed by the numerous new challenges and work overload due to the COVID-19 crisis and consequently reduced staffing levels leading to the prioritization of physical elements of care [11]. Furthermore, the pandemic caused emotional challenges[40] and huge uncertainty about how to react to the pandemic crisis. Still, studies underline that communication rules applied in palliative care should be used [41–43], that healthcare teams need to communicate clinical uncertainty to the relatives[44] to ensure transparent medical care, and to use COVID-19 specific language [45].

### Strengths and limitations of the study

The strengths of this study are the collection of data on end-of-life care of patients, with and without SARS-CoV2 infection, during the first wave of the pandemic in all care settings where people die, the use of validated questionnaires, the adaptation to the pandemic situation, and the assessment of subjective experiences via free-text comments.

A limitation of the study is the focus on the last two days of the patient’s life in many questions. As we adapted the survey from another planned study which used a validated questionnaire we decided to keep the time frame. However, we allowed respondents to provide free-text comments which indicated that bereaved relatives suggest, that a longer period before passing away is relevant.

One limitation might be the relatively small sample size restricting the generalisability of the study. Although we used several recruiting methods, the most successful recruitment way was an interview printed in a regional newspaper that published the survey link. The newspaper has a wide catchment area which includes urban and rural areas and helps to get a wide spectrum of different experiences. Due to the survey’s online format, there was a selection bias resulting in the participation of relatives who felt able to complete an online survey.

## Conclusions

For future pandemics and waves of this pandemic, it is pertinent to reconcile the accompaniment and support of end-of-life patients and their relatives with public health measures. Staying connected with seriously ill and dying patients must be facilitated, allowing face-to-face, (shared) contact whenever possible, and allowing decisions to be made on an individual basis. It should always be possible to visit dying persons. Additionally, video and audio calls between patients and relatives should be offered more widely to support communication and closeness. Furthermore, healthcare professionals play an important role in providing health and psycho-social care in pandemic times, and relatives wish for open, empathetic, and transparent communication to feel included in care decisions – even though having to be physically distant.

## Data Availability

The datasets used and/or analysed during the current study are available from the corresponding author on reasonable request.

## Abbreviations

SARS-CoV2: Severe acute respiratory syndrome coronavirus type 2
PallPan: National Strategy for Palliative Care of Severely Ill and Dying People and their Relatives in Pandemics in Germany
NUM: National Research Network of University Medicine
CHERRIES: Checklist for Reporting Results of Internet E-Surveys
iCODE: International Care Of the Dying Evaluation
PANAS: Positive and Negative Affect Schedule:
